# The effect of statin therapy on the characteristics of coronary artery disease in patients with acute coronary syndrome

**DOI:** 10.1186/1749-8090-8-S1-P152

**Published:** 2013-09-11

**Authors:** D Gulin, J Sikic Vagic, K Kordic, L Vrbanic, E Galic

**Affiliations:** 1 Department of Cardiovascular Disease, University Hospital Sveti Duh, Zagreb, Croatia; 2 University J. J. Strossmayer, Osijek, Croatia

## Background

Statins, in addition to lowering blood lipid levels, also protect the cardiovascular system by improving endothelial dysfunction and producing an anti-inflammatory and anti-thrombotic effect that stabilizes plaques.The aim of this study was to investigate the effect of statin therapy (StTh) on the characteristics of coronary artery disease (CAD) in acute coronary syndrome (ACS).

## Methods

The study included 125 patients who were admitted to Department of Cardiovascular Diseases of University Hospital Sveti Duh for ACS in 2012. An analysis was made of the impact of prior treatment with statins on the leading diagnosis, distribution of the CAD and type of lesion.

## Results

31 patients (24.8%) were treated with StTh prior to admission. Leading diagnoses for patients previously on StTh were STEMI (39%) and unstable angina (39%), while in the group of patients without StTh the leading diagnosis was STEMI (60%). In patients who were treated with StTh the proximal and middle segment of LAD were the most common locations of stenosis. The frequency of single-vessel, two-vessel and three-vessel CAD was equal amongst both groups, while single-vessel CAD was more frequent in patients without prior StTh. According to ACC/AHA classification, type C lesion was discovered in 41% of patients without StTh and in 26% of patients with StTh.

## Conclusion

According to the results of studies, 10-20% of patients experiencing an ACS were being treated with statins prior to the event. In our study, 24.8% of patients had been on StTh. The number of vessels with lesions was seen with equal frequency amongst patients with and without StTh. This apparent lack of benefit can most likely be explained by the increased number of risk factors amongst patients on StTh. Another advantageous finding seen in patients previously treated with statins was a significantly lower proportion of total occlusions, especially STEMI.

